# Return to Work After Breast Cancer Diagnosis and Treatment in Uruguay: Challenges and Determining Factors

**DOI:** 10.7759/cureus.92301

**Published:** 2025-09-14

**Authors:** Natalia Camejo, Cecilia Castillo, Ivan Lyra, Diego Ferreira, Agustin Cabillon, Dahiana Amarillo, Guadalupe Herrera, Lucia Delgado, Gabriel Krygier

**Affiliations:** 1 Oncology Department, Hospital de Clínicas Dr. Manuel Quintela, Montevideo, URY; 2 Oncology Department, Hospital Departamental de Soriano, Soriano, URY; 3 Oncology Department, Sanatorio Mautone, Maldonado, URY; 4 Academic Unit of Preventive and Social Medicine, School of Medicine, University of Uruguay, Montevideo, URY

**Keywords:** adl (activities of daily living), breast neoplasms, cancer survivors, quality of life (qol), return-to-work

## Abstract

Introduction: In Uruguay, breast cancer (BC) is the most common malignancy in women and the leading cause of cancer-related mortality in the female population. It represents a significant public health problem. Improved survival, driven by early diagnosis and therapeutic advances, has brought new challenges related to quality of life, including return to work and domestic activities, which are key to full recovery and social reintegration.

Objective: To determine the rate of return to work in women with BC at least 24 months after diagnosis and to analyze associated factors. To evaluate the impact of diagnosis and treatment on household task performance and to compare quality of life according to work status and domestic performance, using the PROMIS Global Health v1.2 instrument.

Methods: A descriptive longitudinal study was conducted in patients ≥18 years, with stage I-III BC, treated at the Hospital Departamental de Soriano and Sanatorio Mautone. Sociodemographic, clinical, occupational, and domestic data were collected through a structured questionnaire. Quality of life was assessed with PROMIS Global Health v1.2. Chi-square, Fisher’s exact, Mann-Whitney, and Kruskal-Wallis tests were used, with a significance level of 5%.

Results: A total of 128 women were included (median age at diagnosis: 58 years). Among the 74 employed at diagnosis, 60.8% returned to work. Non-return was associated with older age (65 vs. 55 years), lower education, manual labor, mastectomy, axillary dissection, chemotherapy, postoperative complications, arm pain, shoulder limitation, lymphedema, and functional impairment (p<0.05). Among those who returned, 51.1% reported difficulties, mainly physical and due to a lack of adapted tasks; this rose to 79.3% among those who did not return. Regarding domestic activities, 32.5% required greater effort, 32.5% reduced activities, and only 33.3% maintained the same level and effort. Patients who did not return to work or required more effort for household tasks had lower scores in physical health, mental health, global health, and usual activities in PROMIS compared to those maintaining work or domestic tasks without changes (p<0.01).

Conclusions: Return to work and domestic activities in BC survivors are influenced by physical, occupational, and treatment-related factors, and their absence is associated with lower quality of life. Integrating physical rehabilitation, management of sequelae, and vocational guidance into oncology follow-up, especially in resource-limited settings, is essential to promote functional recovery and overall well-being.

## Introduction

Breast cancer (BC) is the most common type of cancer among women worldwide, representing a significant public health challenge [1]. Although incidence rates in North America are higher, with a cumulative risk of 10% before the age of 75 compared to South America (5.6%) and Central America (3.5%), the incidence in young women in Latin America exceeds that of North America, reaching 20% versus 12% [2]. In Uruguay, the cumulative risk is considerably high, with an age-adjusted incidence rate of 75.1 cases per 100,000 women, placing the country among those with the highest incidence in Latin America [1]. These figures make BC one of the main public health concerns in Uruguay, where it is estimated that one in every 11 women will face this disease at some point in their lives. Although incidence rates have remained stable between 2002 and 2019, incidence in women under 45 years of age continues to increase [3].

The increase in BC survival rates, driven by innovations in early diagnosis [4,5], a greater understanding of its biology [6], and advances in treatment [7], has allowed more young women to overcome the disease. However, the rising incidence of BC in this age group is particularly relevant, as it affects their employment opportunities and may lead to long-term social and economic consequences.

One of the most relevant areas for the well-being and quality of life of some patients is the possibility of returning to work after diagnosis and completion of treatment. During the therapeutic process, work attendance is often affected due to treatment-related side effects and the adaptation required by the disease [8]. Employment not only provides an essential source of income but may also offer personal fulfillment. Job loss can negatively impact quality of life, compromising the household’s economic well-being. Furthermore, for many patients, returning to work is seen as a step toward recovery and reintegration into society [9].

According to the review by Tamminga et al. [10], between 56% and 88% of women who survive BC return to work within the first 24 months after diagnosis. However, this proportion varies considerably between countries, influenced by factors such as the health care system, socioeconomic conditions, cultural norms regarding women’s work roles, as well as individual characteristics such as age, type of treatment received, and personal circumstances. A study conducted in Uruguay revealed that only 50% of patients who were employed before the diagnosis of BC were able to resume their work activity two years after treatment. Moreover, 68.6% faced difficulties in returning to work, and 83.3% experienced problems with household tasks [11].

Finally, it should be noted that returning to work, although generally promoted as a desirable objective, does not necessarily represent the best option for all patients. The cancer experience may lead some women to reassess their priorities and redefine their life projects, including their relationship with the work environment. In this regard, there are still few studies exploring whether resuming work activity after oncologic treatment effectively translates into a better quality of life.

This study aimed to describe the employment situation and domestic workload in a cohort of Uruguayan female BC survivors, as well as to explore their relationship with indicators of physical and emotional well-being, with the objective of generating local evidence to contribute to a better understanding of the challenges these patients face in their daily lives.

## Materials and methods

Objective

The aims of this study were to determine the rate of return to work among women with BC at least 24 months after diagnosis, to analyze the factors associated with return to work, to assess the effect of BC on the performance of household tasks, and to compare overall quality of life, measured with the PROMIS Global Health v1.2 instrument, according to employment status and household performance.

Materials and methods

A descriptive, cross-sectional study was conducted, including women over 18 years of age diagnosed with stage I to III BC, sequentially recruited from patients under follow-up at the Oncology Services of the Hospital Departamental de Soriano and the Sanatorio Mautone. Eligibility required a minimum of 24 months since diagnosis, ensuring adequate time to assess return-to-work outcomes and reducing recall bias.

The Hospital Departamental de Soriano, located in the Department of Soriano, is a public healthcare center, while the Sanatorio Mautone, located in the Department of Maldonado, is a private healthcare institution. Both centers are teaching affiliates of the Hospital de Clínicas “Dr. Manuel Quintela” in Montevideo, the main university hospital in Uruguay. In both institutions, oncology care and academic training are provided, which allowed us to access a diverse patient population for this study.

Participants completed a structured ad hoc questionnaire, developed by the investigators through consensus and informed by previous literature and clinical experience, addressing sociodemographic variables, employment status, and household task performance during the three months prior to diagnosis and at least 24 months afterward, as well as employer support received. Although not previously validated, this questionnaire was specifically designed for the present study to complement the PROMIS Global Health v1.2 instrument (Table 6 of Appendices).

For the analysis of changes in employment activity and household task performance, five categories were defined by consensus among the investigators: no change, where patients remained employed with the same number of working hours at both time points in the questionnaire or performed the same household tasks with similar effort; reduced workload or functional capacity, where patients remained employed but worked fewer hours, performed the same number of household tasks with greater effort, or performed fewer tasks at the second time point; no return to work, where patients employed at the time of diagnosis were not working at the second time point (due to unemployment, early retirement, or pension); emergent employment, where patients unemployed or unable to work at the time of diagnosis were employed at the time of the survey; and sustained unemployment or functional inactivity, where patients were unemployed, retired, or did not perform household tasks at either time point.

In addition, the PROMIS Global Health v1.2 questionnaire, Spanish version (Latin America), which is freely accessible and officially validated, was administered to assess overall quality of life through its physical health and mental health domain scores. This instrument was selected because it is validated for use in Latin America, provides standardized T-scores referenced to the general population, and offers a concise yet comprehensive alternative to longer instruments such as the SF-36 [12].

Statistical analysis

Eligible patients were included sequentially. Variables were categorized as qualitative or quantitative. Qualitative variables are presented using absolute and relative percentage frequencies. Quantitative variables are presented as mean and standard deviation in the case of normal distribution, and as median and IQR in the case of non-normally distributed data.

Normality of continuous variables was assessed using the Shapiro-Wilk test and visual inspection of histograms and Q-Q plots. The association of sociodemographic, disease, treatment, and work-related characteristics with employment status before and >24 months after diagnosis was evaluated using Pearson’s Chi-square test for categorical variables; when expected cell counts were <5, Fisher’s exact test was applied. For continuous variables not meeting the assumption of normality, comparisons between two independent groups were performed with the Mann-Whitney U test.

T-scores for the physical and mental domains of the PROMIS Global Health v1.2 questionnaire were calculated according to the instrument guidelines. The association between PROMIS v1.2 scale scores and household activities was assessed using the Kruskal-Wallis test. All tests were two-sided with a significance level of 5%. Analyses were performed using IBM SPSS Statistics, version 18.0 (IBM Corp., Armonk, NY, USA).

Ethical considerations

The study was approved by the relevant ethics committee (approval number: 158-23) and was conducted in accordance with the Declaration of Helsinki, MERCOSUR regulations, and the research guidelines of the National Ethics Committee (2019). Written informed consent was obtained from all participants, and data confidentiality was ensured in accordance with Uruguay’s Personal Data Protection Law (Law No. 18.331).

## Results

Clinical and sociodemographic data

A total of 128 patients diagnosed with early-stage BC (stages I-III) were included. The median age at diagnosis was 58 years (IQR 34-80), and 27% of patients had dependent children. Thirty-one percent lived alone, and more than half (52%) had completed secondary education or higher. The remaining epidemiological and demographic characteristics are shown in Table 1.

**Table 1:** Epidemiological and demographic characteristics at diagnosis and stage of patients included in the study (n=128) ¹Median (min-max); the rest are presented as n (%)

Characteristics	Total, N=128	I/II, N=105	III, N=23
Institution			
Hospital Departamental de Soriano	100 (78%)	80 (76%)	20 (87%)
Sanatorio Mautone	28 (22%)	25 (24%)	3 (13%)
Age at diagnosis¹	58.0 (37.0-87.0)	58.0 (37.0-87.0)	54.0 (43.0-79.0)
Current age¹	62.0 (34.0-90.0)	63.0 (34.0-90.0)	58.0 (40.0-82.0)
Rural residence			
No	117 (92%)	95 (91%)	22 (96%)
Yes	10 (7.9%)	9 (8.7%)	1 (4.3%)
Marital status			
Married/cohabiting	64 (50%)	49 (47%)	15 (65%)
Divorced/separated	27 (21%)	22 (21%)	5 (22%)
Single	8 (6.3%)	7 (6.7%)	1 (4.3%)
Widowed	29 (23%)	27 (26%)	2 (8.7%)
Number of children¹	2.0 (0.0-6.0)	2.0 (0.0-6.0)	2.0 (0.0-4.0)
Has minors under care			
No	93 (73%)	79 (75%)	14 (61%)
Yes	35 (27%)	26 (25%)	9 (39%)
Lives alone			
No	87 (69%)	68 (65%)	19 (83%)
Yes	40 (31%)	36 (35%)	4 (17%)
Lives with a partner			
No	59 (46%)	52 (50%)	7 (32%)
Yes	68 (54%)	53 (50%)	15 (68%)
Lives with children			
No	71 (60%)	59 (61%)	12 (55%)
Yes	47 (40%)	37 (39%)	10 (45%)
Lives with parents			
No	107 (84%)	87 (84%)	20 (87%)
Yes	20 (16%)	17 (16%)	3 (13%)
Contributes to household expenses			
No	31 (24%)	25 (24%)	6 (27%)
Yes	96 (76%)	80 (76%)	16 (73%)
Educational level			
Primary/incomplete secondary	61 (48%)	52 (50%)	9 (39%)
Completed secondary/technical school (UTU)/university	67 (52%)	53 (50%)	14 (61%)

Regarding treatment characteristics, 60% of patients received chemotherapy, and 75% received radiotherapy. Axillary lymph node dissection was performed in 71%, and mastectomy in 48%. Only 16% underwent breast reconstruction. The remaining data are detailed in Table 2.

**Table 2:** Disease-free interval, surgical characteristics, and oncologic treatments according to stage at diagnosis (n=128) ¹Median (range) for continuous variables; absolute value and percentage for categorical variables.

Variable	Total, N=128¹		I/II, N=105¹	III, N=23¹
Disease-free interval (months)¹		33.0 (24.0-1496.0)	33.0 (24.0-1496.0)	34.0 (25.0-119.0)
Chemotherapy				
No		51 (40%)	50 (48%)	1 (4.3%)
Yes		76 (60%)	54 (52%)	22 (96%)
Type of breast surgery				
Breast-conserving surgery		66 (52%)	58 (55%)	8 (35%)
Mastectomy		62 (48%)	47 (45%)	15 (65%)
Type of axillary surgery				
Sentinel lymph node biopsy		36 (29%)	36 (35%)	0 (0%)
Axillary lymph node dissection		90 (71%)	67 (65%)	23 (100%)
Breast reconstruction				
No		106 (84%)	88 (84%)	18 (86%)
Yes		20 (16%)	17 (16%)	3 (14%)
Radiotherapy				
No		32 (25%)	29 (28%)	3 (13%)
Yes		96 (75%)	76 (72%)	20 (87%)
Hormone therapy				
No		13 (10%)	9 (8.7%)	4 (17%)
Yes		114 (90%)	95 (91%)	19 (83%)
Biological therapy				
No		108 (85%)	90 (86%)	18 (82%)
Yes		19 (15%)	15 (14%)	4 (18%)

Return to work and performance in household tasks

Regarding employment status, 54 women (42.2%) were unemployed at the time of diagnosis; among these, only 3 (0.6%) were working 24 months after diagnosis. Among the 74 women who were employed prior to diagnosis, 45 (60.8%) returned to work at the same workplace, while 39.2% did not resume employment.

Among those who returned to work, 51.1% reported having experienced difficulties, the most frequent being physical limitations in performing the same task (n=13, 56.5%), lack of availability of adapted tasks (n=9, 39.1%), and, to a lesser extent, emotional causes. In 43.5% of cases (n=10), more than one of these causes was reported.

In the group that did not return to work (n=29), 79.3% (n=23) reported physical difficulties in performing their usual tasks, and 62% (n=18) mentioned both physical difficulties and the lack of alternative tasks. Only one patient reported emotional difficulties as the main barrier.

In the bivariate analysis of the 74 patients who were employed at the time of diagnosis, those who did not return to work were significantly older (median age: 65 vs. 55 years, p<0.001), more frequently engaged in manual labor (72% vs. 36%, p=0.004), and had a higher proportion of mastectomy (66% vs. 36%, p=0.017), axillary dissection (90% vs. 67%, p=0.046), chemotherapy (74% vs. 26%, p=0.028), postoperative complications (28% vs. 6.7%, p=0.020), arm pain (90% vs. 44%, p<0.001), shoulder movement limitation (76% vs. 47%, p=0.016), lymphedema (41% vs. 8.9%, p=0.001), and physical limitation (90% vs. 58%, p=0.004). These findings are summarized in Table 3.

**Table 3:** Clinical, sociodemographic, and functional characteristics of women employed at the time of diagnosis, according to employment status at 24 months (n=74) ¹Median (min-max); n (%). ²Wilcoxon rank-sum test; Fisher’s exact test.

Total, N=74¹		Employed before and after diagnosis, N=45¹	Employed before and not employed after diagnosis, N=29¹	p-value²
Current age	58.0 (34.0-80.0)	55.0 (34.0-73.0)	65.0 (42.0-80.0)	<0.001
Marital status				
Married/cohabiting	40 (54%)	25 (56%)	15 (52%)	0.8
Divorced/separated	20 (27%)	12 (27%)	8 (28%)	0.8
Single	7 (9.5%)	5 (11%)	2 (6.9%)	0.8
Widowed	7 (9.5%)	3 (6.7%)	4 (14%)	0.8
Educational level				
Primary/incomplete secondary	25 (34%)	12 (27%)	13 (45%)	0.2
Completed secondary/technical school (UTU)/university	49 (66%)	33 (73%)	16 (55%)	0.2
Stage				
I/II	59 (80%)	34 (76%)	25 (86%)	0.4
III	15 (20%)	11 (24%)	4 (14%)	0.4
Manual labor (pre-diagnosis job)				
No	37 (50%)	29 (64%)	8 (28%)	0.004
Yes	37 (50%)	16 (36%)	21 (72%)	0.004
Surgery on the dominant hand side				
No	38 (51%)	25 (56%)	13 (45%)	0.5
Yes	36 (49%)	20 (44%)	16 (55%)	0.5
Type of breast surgery				
Breast-conserving surgery	39 (53%)	29 (64%)	10 (34%)	0.017
Mastectomy	35 (47%)	16 (36%)	19 (66%)	0.017
Type of axillary surgery				
Sentinel lymph node biopsy	17 (24%)	14 (33%)	3 (10%)	0.046
Axillary lymph node dissection	55 (76%)	29 (67%)	26 (90%)	0.046
Chemotherapy				
No	19 (26%)	7 (16%)	12 (41%)	0.028
Yes	54 (74%)	37 (84%)	17 (59%)	0.028
Radiotherapy				
No	13 (18%)	5 (11%)	8 (28%)	0.12
Yes	61 (82%)	40 (89%)	21 (72%)	0.12
Hormone therapy				
No	8 (11%)	5 (11%)	3 (11%)	>0.9
Yes	65 (89%)	40 (89%)	25 (89%)	>0.9
Biological therapy				
No	59 (81%)	35 (80%)	24 (83%)	>0.9
Yes	14 (19%)	9 (20%)	5 (17%)	>0.9
Complications				
No	63 (85%)	42 (93%)	21 (72%)	0.02
Yes	11 (15%)	3 (6.7%)	8 (28%)	0.02
Scar pain				
No	50 (68%)	32 (71%)	18 (62%)	0.5
Yes	24 (32%)	13 (29%)	11 (38%)	0.5
Arm pain				
No	28 (38%)	25 (56%)	3 (10%)	<0.001
Yes	46 (62%)	20 (44%)	26 (90%)	<0.001
Shoulder range-of-motion limitation				
No	31 (42%)	24 (53%)	7 (24%)	0.016
Yes	43 (58%)	21 (47%)	22 (76%)	0.016
Lymphedema				0.001
No	58 (78%)	41 (91%)	17 (59%)	0.001
Yes	16 (22%)	4 (8.9%)	12 (41%)	0.001
Physical limitation				0.004
No	22 (30%)	19 (42%)	3 (10%)	0.004
Yes	52 (70%)	26 (58%)	26 (90%)	0.004
Required psychological support				
No	64 (86%)	40 (89%)	24 (83%)	0.5
Yes	10 (14%)	5 (11%)	5 (17%)	0.5
Required psychotropic medication				
No	64 (88%)	41 (91%)	23 (82%)	0.3
Yes	9 (12%)	4 (8.9%)	5 (18%)	0.3
Duration of medication (months)	12.0 (2.0-24.0)	10.0 (4.0-24.0)	12.0 (2.0-24.0)	>0.9

Regarding household chores, 33.3% (n=42) reported performing them with the same effort as before the diagnosis, another 32.5% (n=41) with greater effort, and 32.5% (n=41) reported doing fewer tasks. Only two women (1.6%) reported being unable to perform any household chores.

Quality of life and global functioning according to employment status and household performance

Regarding quality of life, 98 patients completed the PROMIS Global Health v1.2 questionnaire. Women who remained employed showed better scores in physical health (median IQR 39.8 vs. 37.4; p=0.003), mental health (p=0.011), global health (p=0.010), and usual activities (p=0.010) compared to those who did not return to work (Table 4).

**Table 4:** Distribution of scores on the PROMIS Scale v1.2 Global Health according to pre- and post-diagnosis employment status ¹Median (IQR). ²Wilcoxon rank sum test.

Work activity				
	Total, N=74¹	Employed before and after diagnosis, N=45¹	Employed before diagnosis and unemployed after, N=29¹	p-value²
T score, physical health	37.4 (29.6-67.7)	39.8 (37.4-67.7)	37.4 (29.6-67.7)	0.003
T score, mental health	43.5 (33.8-67.6)	43.5 (36.3-67.6)	43.5 (33.8-67.6)	0.011
Global health score	3.0 (2.0-5.0)	3.0 (3.0-5.0)	3.0 (2.0-5.0)	0.01
Usual activities score	3.0 (2.0-5.0)	3.0 (3.0-5.0)	3.0 (2.0-5.0)	0.01

**Figure 1:**
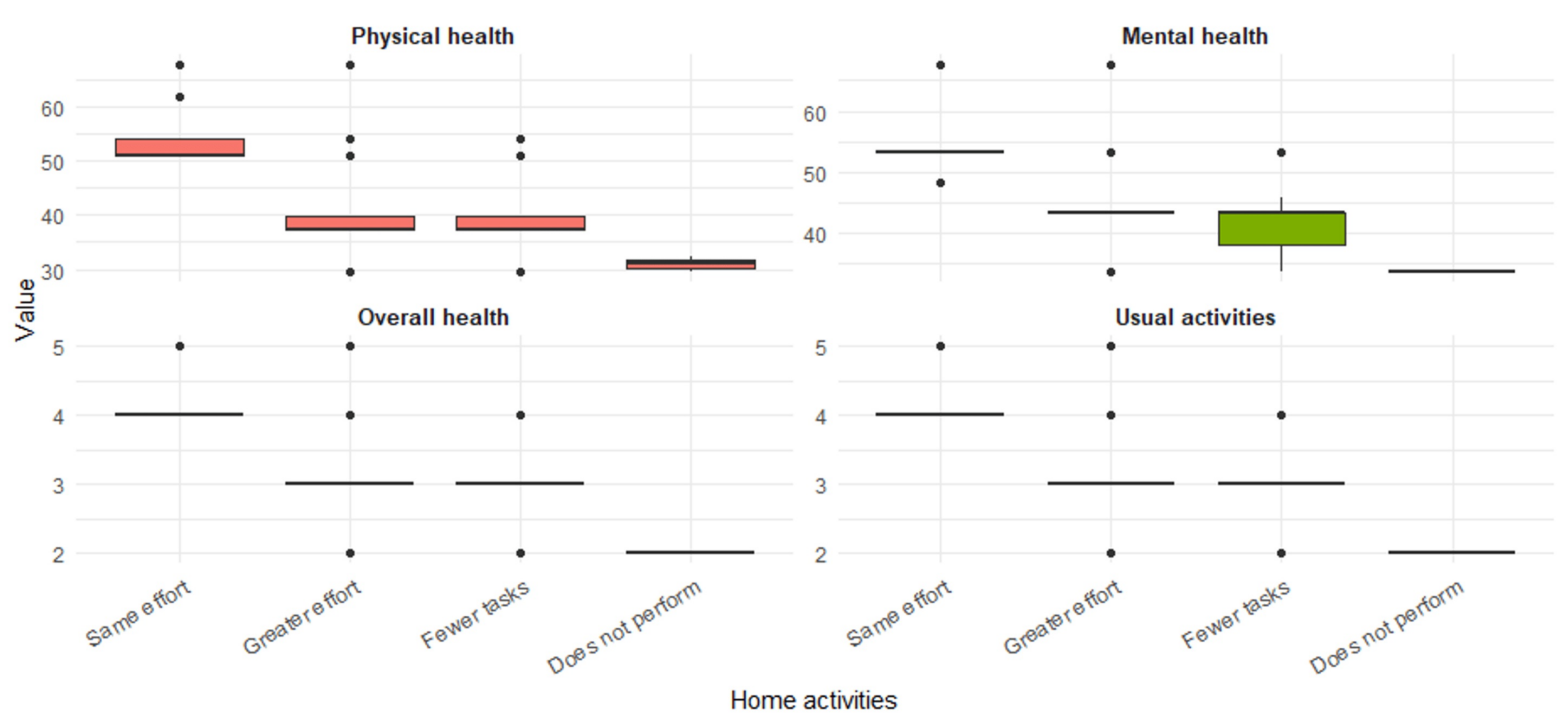
Results of the PROMIS Scale v1.2 Global Health according to household activity in 98 women

The PROMIS Global Health v1.2 questionnaire scores showed significant differences according to the level of household activity reported by the patients (Figure 1). Those who reported performing the same tasks as before diagnosis and with the same effort obtained the best results in all evaluated domains: physical health (median 50.8), mental health (53.3), global health (4.0), and usual activities (4.0). In contrast, patients who were unable to perform household tasks had the lowest scores in all domains: physical health (31.0), mental health (33.8), global health (2.0), and usual activities (2.0). Women who performed tasks with greater effort or in smaller amounts also showed intermediate scores, with statistically significant differences in all evaluated items (p<0.001) (Table 5; Figure 1 ).

**Table 5:** Results of the PROMIS Scale v1.2 Global Health according to household activity in 98 women ¹Median (min-max). ²Kruskal-Wallis rank sum test.

Total, N=98¹		Unable to perform them, N=21	Performs the same tasks as before diagnosis with the same effort, N=28	Performs the same tasks as before diagnosis with greater effort, N=34	Performs fewer tasks than before, N=34	p-value²
T score, physical health	39.8 (29.6-67.7)	31.0 (29.6-32.4)	50.8 (50.8-67.7)	37.4 (29.6-67.7)	37.4 (29.6-54.1)	<0.001
T score, mental health	43.5 (33.8-67.6)	33.8 (33.8-33.8)	53.3 (48.3-67.6)	43.5 (33.8-67.6)	43.5 (33.8-53.3)	<0.001
Global health score	3.0 (2.0-5.0)	2.0 (2.0-2.0)	4.0 (4.0-5.0)	3.0 (2.0-5.0)	3.0 (2.0-4.0)	<0.001
Usual activities score	3.0 (2.0-5.0)	2.0 (2.0-2.0)	4.0 (4.0-5.0)	3.0 (2.0-5.0)	3.0 (2.0-4.0)	<0.001

**Figure 2:**
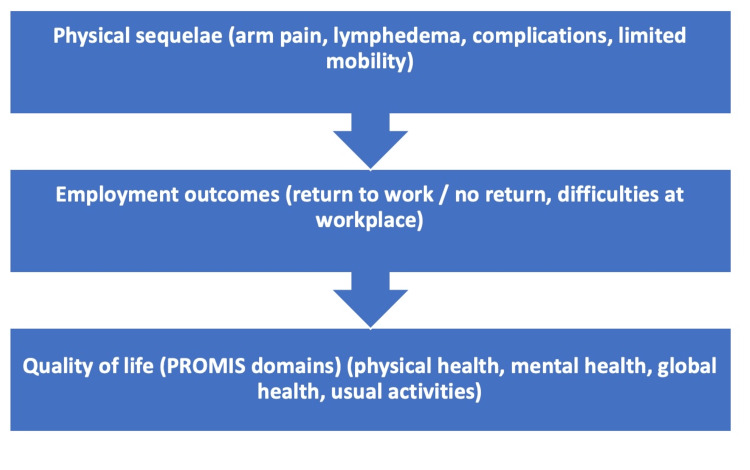
Associations between physical sequelae, employment outcomes, and quality of life Note: Arrows represent associations, not causal pathways.

Figure 2 summarizes the conceptual relationships identified in this study between employment status, household task performance, physical sequelae, and quality of life.

## Discussion

Understanding the factors that influence returning to work and resuming household tasks in women who have experienced BC is key to improving their quality of life and promoting comprehensive recovery. Based on the data obtained from this cohort of Uruguayan patients, it was possible to identify specific barriers that hinder the return to work and the full performance of household tasks, as well as their impact on physical and emotional well-being.

A total of 128 women were included, with a median age at diagnosis of 58 years. Most lived in urban areas, had a medium or high educational level, and 76% contributed in some way to household expenses. Regarding treatment, 60% received chemotherapy, 75% radiotherapy, 48% underwent mastectomy, and 71% axillary dissection. The disease-free interval was 33 months.

The return-to-work rate observed in our cohort (60.8%) was lower than that reported in Europe and North America, where, although rates range between 56% and 88%, most studies report figures around 70-80% two years after diagnosis [10,13,14]. In Asia, the information is more heterogeneous, with figures varying widely, from 43% to 93%, with a trend toward lower rates in countries with lower economic development or in populations with a higher proportion of manual labor jobs [15]. In Latin America, although data are more limited, a previous study in Uruguay showed that only 50% of patients return to work [11], and an observational Brazilian study reported a return-to-work rate of 60.4% at 24 months after diagnosis [16], figures similar to those found in our study and comparable to those of low-income populations in the United States [17].

Patients who did not return to work had a significantly higher median age and were more frequently engaged in manual labor compared to those who did return, which is consistent with international reviews [10] and meta-analyses [16]. However, unlike what has been described in the literature, where a lower educational level is often associated with a lower probability of returning to work, our cohort did not show significant differences in this regard between the two groups [10,16].

Although a more advanced stage is traditionally associated with a lower rate of return to work, due to greater disease burden and more aggressive treatments, our study did not find significant differences in this aspect [17]. Regarding treatment, patients who did not return to work had undergone more aggressive approaches, with higher rates of mastectomy, axillary dissection, and chemotherapy. They also presented more postoperative complications, arm pain, shoulder movement limitation, lymphedema, and physical restriction, which is in line with the literature [18], where these musculoskeletal sequelae are associated with reduced functional capacity and difficulties in performing work activities.

Several studies have identified that work-related factors such as the nature of the job (physical vs. administrative), the possibility of workplace accommodations, company size, and prior job stability significantly influence the return to work after illness [17,19]. Our results are consistent with this evidence: among those who managed to return to work, 51.1% reported having faced difficulties, mainly physical (56.5%) and due to the lack of adapted tasks (39.1%). In the group that did not return to work, these barriers were even more pronounced, being reported by 79.3% and 62% of patients, respectively. This reinforces the decisive role of working conditions and workplace flexibility in functional recovery and reintegration. Regarding its impact on quality of life, patients who continued working after diagnosis showed better scores in physical, mental, global health, and usual functions, supporting a positive association between employment and well-being. These findings are consistent with the literature, which links lack of return to work with greater pain, fatigue, psychological symptoms, and functional decline [20-24]. Job loss can reflect or exacerbate physical and emotional sequelae, as well as create economic and social difficulties by reducing daily structure and social support [21,24]. Longitudinal studies have shown that employment acts as a protective factor during the first years after diagnosis [20,22].

Our results show that two-thirds of patients experienced difficulties in resuming household tasks after treatment: 32.5% reported requiring greater effort, and another 32.5% performed fewer activities. This is consistent with what has been reported in the literature, where 64.6% of survivors cite fatigue and exhaustion as the main barriers to domestic activities [25]. Furthermore, prospective studies have documented that household activity levels significantly decrease during the first year after surgery and generally do not return to preoperative levels within that period [26]. Even among those who manage to resume activities, many do so at a slower pace or with greater physical effort, as evidenced in our study [26-28]. In our cohort, patients who resumed household tasks with the same effort as before diagnosis showed better quality of life across all evaluated domains, whereas those with greater limitations reported significant deterioration. These findings align with the available evidence, which consistently indicates that limitations in instrumental activities of daily living, such as household tasks, are associated with poorer quality of life in various populations, including women with chronic and oncological diseases [29,30]. In particular, restriction in these activities has been shown to be related to a progressive decline in physical, psychological, and social well-being [30]. Moreover, several studies have identified functional difficulty in daily tasks as one of the main determinants of health-related quality of life deterioration, even above symptoms such as pain [29,30]. Taken together, these data underscore the need to consider the impact of BC not only on formal employment but also on unpaid household activities, which represent a substantial part of patients’ well-being and functionality. These findings contribute to a better understanding of the interplay between employment, household tasks, and quality of life in BC survivors. However, some limitations of this study must also be acknowledged.

The descriptive, cross-sectional design does not allow causal inferences to be established, and the relatively limited sample size reduces the statistical power of some comparisons. Furthermore, the study was conducted in only two centers within a single country, which restricts the generalizability of the findings. Another limitation is the use of self-reported questionnaires, which may be subject to recall and information bias. Despite these limitations, the inclusion of validated instruments such as the PROMIS Global Health v1.2 and the focus on both employment and household tasks provide valuable insights into the survivorship experience of Latin American women with BC.

## Conclusions

The findings of this study show that, although a significant proportion of BC patients are able to resume their work or domestic activities after completing oncological treatments, multiple challenges persist that affect their quality of life and overall well-being. Factors such as age, type of previous occupation, having received more aggressive treatments, and presenting greater physical sequelae significantly influence both work reintegration and perceived overall health. In this context, it is essential to implement follow-up and multidisciplinary support strategies that address not only disease control but also accompaniment throughout the process of social and occupational reintegration. Actively addressing physical rehabilitation, fatigue management, persistent pain, and psychosocial aspects will improve the quality of life of survivors and promote comprehensive recovery. Our results reinforce the need to incorporate rehabilitation and vocational guidance programs into oncology services, particularly in resource-limited settings.

## Appendices

**Table 6:** Questionnaire on return to work after breast cancer diagnosis and treatment

Treatment: Biological therapies	Yes/No
Age	Numeric
Menopausal status	Premenopausal/Postmenopausal
Where do you live?	Interior/Montevideo
Rural area	Yes/No
Marital status	Married/In a relationship/Divorced/Separated/Single/Widowed
Do you have children?	Yes/No
How many children?	Numeric
Are they minors?	Yes/No
Who do you live with?	Alone/With partner/With children/With parents
Are you head of household?	Yes/No
Educational level	No schooling/Primary (complete/incomplete)/Secondary (complete/incomplete)/Technical school (UTU)/Tertiary
What is your job?	Public administration/Commerce/Construction/Industry/Domestic services/Business services/Other services
Self-employed?	Yes/No
How many hours do you work	≥35 hours/week/<35 hours/week
Did you receive employer support after diagnosis?	Yes/No
Were your tasks adapted after diagnosis?	Yes/No/Not necessary
Did you receive support from your partner after diagnosis?	Yes/No
Are you a housewife?	Yes/No
Do you perform repetitive movements with upper limbs?	Yes/No
Do you lift/carry weight?	Yes/No
Do you maintain uncomfortable postures?	Yes/No
Did you receive psychological/psychiatric treatment?	Yes/No
Did you take medication for distress, anxiety, or sleep?	Yes/No
If yes, which medication?	Free text
For how long?	Free text
Is your dominant hand the same as the operated side?	Yes/No
Current complications: Pain in scar area	Yes/No
Current complications: Pain in arm on surgery side	Yes/No
Current complications: Limited shoulder mobility	Yes/No
Current complications: Lymphedema	Yes/No
Current complications: Physical limitation from treatments	Yes/No
